# Urinary Estradiol in Captive Bonobos: Variation With Reproductive State and Sexual Swelling

**DOI:** 10.1002/ajp.70041

**Published:** 2025-05-14

**Authors:** Sara Cotton, Klaree Boose, Sedona Espstein, Audra Meinelt, Josh Snodgrass, Frances White

**Affiliations:** ^1^ Department of Anthropology University of Oregon Eugene Oregon USA; ^2^ Eugene Police Department Eugene Oregon USA; ^3^ Columbus Zoo and Aquarium Powell Ohio USA

**Keywords:** apes, endocrinology, estradiol, reproduction, sexual swelling

## Abstract

Estradiol is known to have a variety of biological and behavioral effects, but monitoring its function is complex given the many factors influencing its variation. This necessitates large sample sizes which are challenging in captive and wild situations. This study validates the use of opportunistically collected urinary estradiol levels (E2) for use in reproductive monitoring and behavioral research in bonobos (*Pan paniscus*). We analyzed frozen urine samples from four Columbus Zoo adult females over 4 years for estradiol and creatinine concentrations (*n* = 117). While E2 was significantly higher in pregnant versus nonpregnant females (F = 66.30, *df* = 1, *p* < 0.001) it was not significantly different between lactating and regularly cycling females (F = 0.40, *df* = 1, *p* = 0.5304). Among the regularly cycling females, there was a significant positive regression between E2 and sexual swelling size (F = 4.43, df = 1, 81, *p* = 0.0384). No differences in E2 variation were detected between individuals in this study. Specifically, when the amount of variation in estradiol due to sexual swelling was statistically controlled for, there was no significant effect of age (*n* = 83, r = 0.08059, *p* = 0.4689) or rank (*n* = 83, r = 0.1361, *p* = 0.22) on estradiol variation. Overall, these findings indicate that opportunistically sampled urinary estradiol can be paired with visual observation to help detect changes in reproductive status. The shift from lactational amenorrhea back to estrogen cycling may be less clearly defined than expected, and uneven sampling may exacerbate difficulty in detecting some of the more subtle shifts in estradiol levels. While it is known that extended maximal tumescence in bonobos may function to obscure the exact date of ovulation, we did confirm that ratings of visual tumescence still provide useful information regarding relative estradiol levels. By publishing more methodologies and results of this kind, we hope to promote the continued study of estradiol in bonobos as it is relevant to both health monitoring and behavioral research goals.

## Summary

- Measurement of excreted estradiol levels in urine is a valid means of detecting expected estrogen trends in bonobos, and can be used for reproductive monitoring purposes or behavioral endocrinology research.
- While urinary estradiol levels were significantly different between pregnant and nonpregnant female bonobos, it was not possible to differentiate between lactating and regularly cycling females with opportunistic sampling.
- Significant overlaps in urinary estradiol values between reproductive statuses indicate that single hormone values should not be used in isolation to determine reproductive status and irregularly sampled data sets should be carefully and contextually interpreted.
- Urinary estradiol levels significantly correlated with increases in keeper‐reported sexual swelling ratings in captive female bonobos; these ratings still provide useful information regarding relative estradiol levels and ovulatory status, despite knowledge that extended maximal tumescence may serve to obscure the exact date of ovulation.

## Graphical abstract

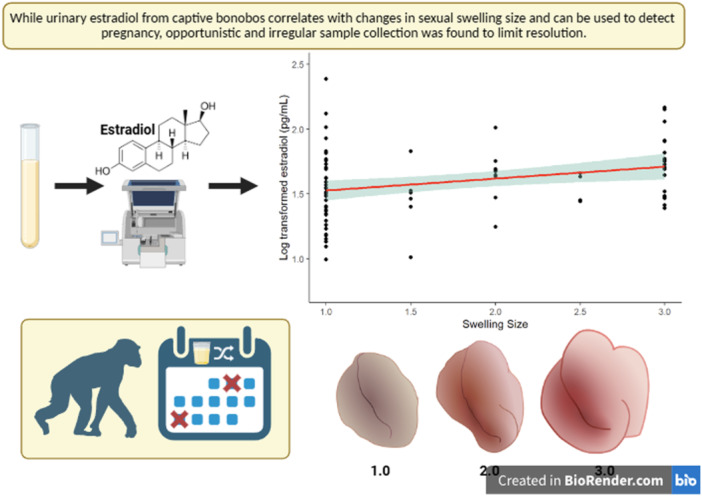

## 1 Introduction

Various urinary metabolites of estrogen hormones have been validated as reliable, noninvasive means of monitoring ovarian function and are often measured in primate endocrinology studies to track ovulation and to detect pregnancy (Czekala et al. 2017; Durgavich et al. 2022; Emery Thompson 2005; Heistermann et al. 1996; Jurke et al. 2001). Estrogens, in human and nonhuman primates alike, exhibit cyclic peaks with the female ovarian monthly cycle with levels rising across the follicular phase, peaking around ovulation, and decreasing over the luteal phase (Graham 2012). In addition to exhibiting cyclicity with monthly female ovarian cycle levels, estrogen levels vary significantly over the course of pregnancy with levels rising steadily until parturition, dramatically dropping off after birth, and remaining low through the lactational phase until females resume sexual receptivity and cycles return (Graham 2012; Hashimoto et al. 2022; Saltzman et al. 2011). Age is known to affect estrogen levels in primates as regular cycling does not occur fully until after puberty, reaches a peak in midlife, and steadily declines until cessation during menopause (Graham 2012; Saltzman et al. 2011; Wood et al. 2023).

There are three main endogenous estrogens—estrone (E1) and estriol (E3) are known as weak estrogens and are only present in high quantities during pregnancy and menopause, while estradiol (E2) is the predominant estrogen in naturally cycling females of all terrestrial vertebrates and fish (Baker 2013). Circulating levels of serum estradiol are the gold standard for estimating estradiol levels in the body and are, therefore, most often measured in laboratory studies of reproductive socio‐endocrinology. As access to regular bloodwork and sampling is rare, however, those interested in studying estrogen cycling noninvasively in more naturalistic settings have developed methodologies for studying correlates of serum estradiol in urine (Brown et al. 2004; Coburn et al. 2019).

Urinary conjugates of estrone have relatively high correlation with serum estradiol levels and have therefore been used to predict ovarian cyclicity (Coburn et al. 2019; Munro et al. 1991). Urinary conjugates are circulating metabolites of endogenous estrogens distributed throughout the body and are thus only indirectly tied to circulating estradiol levels with an imperfect correlation value dependent upon rates of excretion, metabolism, and methodological differences. While the actual levels of excreted estradiol (17β‐estradiol) present in the urine may similarly not be a perfect analog for changes in serum estradiol, they are known to be moderately correlated with serum estradiol and their excretion rate may reflect longer term changes in circulating steroid levels (Coburn et al. 2019; Ross and French 2011; Stanczyk et al. 1980).

Biologically, estradiol contributes to the maintenance of reproductive processes and sexual differentiation in females across species. Estradiol is also known to be involved in the regulation of a number of behaviors including female sexual initiation and motivation behaviors as well as perceptions of female attractiveness (Wallen 1990; Zehr et al. 1998). Studying female sexual motivation becomes particularly relevant in species in which sexual acts are not tied to a mating season or hormonal stimulation as female motivation becomes the primary regulator of mating (Wallen 1990). There is also evidence that estradiol has a role in regulating female‐female aggressive behaviors in stump‐tailed macaques (*Macaca arctoides*) (Cerda‐Molina et al. 2022; Michael and Zumpe 1993) and Wied's black‐tufted ear marmosets (*Callithrix kuhli*) (Ross and French 2011). Estradiol treatments have been seen to increase affective responses to threats in post‐menopausal female rhesus macaques (*Macaca mulatta*) (Bliss‐Moreau and Baxter 2018), and to potentially regulate general levels of affiliation and pro‐social behaviors (Michopoulos et al. 2011). The regulatory effects of estradiol on behavior seem to vary between individuals, most often based on social status (Zumpe and Michael 1989; Reding et al. 2020; Michopoulos et al. 2011).

Bonobos (*Pan paniscus*) form a particularly interesting model with which to study estrogen and its potential socio‐sexual functions, due both to their evolutionary closeness to humans, and their high expression of unique female sexual behaviors. Both humans and bonobos practice extended sexuality outside of the context of reproduction and bonobos can have long interbirth intervals with extended infant investment periods (Hashimoto et al. 2022). In captivity, adolescent female bonobos have been seen to begin menstruating between 5 and 9 years of age and have their first pregnancy between 11 and 13 years of age (Thompson‐Handler 1990; Vervaecke et al. 1999). Average menstrual cycle length for bonobos appears to range between 30 and 50 days with an average of 33.8 days reported in captivity and 40.8 in the wild (Douglas et al. 2016; Heistermann et al. 1996). Bonobo interbirth intervals in the wild range between three and half to 6 years with a weaning period ranging between 3 and half to 5 years (Hashimoto et al. 2022). Bonobos exhibit lactational amenorrhea, or the disruption of normal menstrual cycling, for about 24 months following birth, despite resuming sexual activities just 6 months after birth (Hashimoto et al. 2022). On occasion, bonobos are capable of giving birth after relatively short time intervals and have been seen to nurse two offspring simultaneously (Furuichi and Hashimoto 2002). Given the extended, often multi‐year, periods of postpartum infertility in bonobos, much of their lives are spent in various nonovulatory phases of estrogen fluctuation. It is important that we develop an understanding of the effects of these various reproductive stages on estrogen levels if we are to correctly interpret fluctuations in estrogen levels.

Like many primates, bonobos exhibit highly visible sexual swellings with lengthy periods of maximum tumescence (Douglas et al. 2016; Thompson‐Handler 1990). Many theories have been proposed as to the function of extended tumescence in primates. However, sexual swellings of female bonobos are generally regarded as poor determinants of the timing of ovulation (Douglas et al. 2016; Reichert et al. 2002), thus highlighting the importance of hormonal monitoring as a means of tracking ovulation and fertility.

Only a few studies have attempted to characterize the endocrine pattern of ovarian cycles in captive bonobos by measuring various hormones present in both urinary and fecal samples. The earliest of these studies managed to pool large‐scale sampling of ovarian hormones across captive bonobo populations to establish hormone profiles, estimates of ovarian cycle length, and compare ovulatory timing with sexual swellings and sexual behaviors in bonobos (Heistermann et al. 1996; Jurke et al. 2001; Reichert et al. 2002). The trends identified in these early characterizations of bonobo endocrine function have generally been replicated by subsequent data from wild bonobo populations (Douglas et al. 2016; Hashimoto et al. 2022). A summary of these results can be reviewed in Table 1 below.

**Table 1:** A brief summary of previous studies monitoring female reproductive endocrinology in bonobos.

Study	Hormones measured	Methodology used	Primary findings
Heistermann et al. (1996)	E1C and PdG	EIA Urine and fecal samples 5‐7 days/week Captive	Hormone profiles of urinary E1C are better than those of fecal E1C High correlation between urinary and fecal PdG Cycle lengths calculated (31–37 days)
			Variability occurs in follicular phase
			Diagnostic value of E1C levels in early pregnancy detection
			E1C peak does occur during maximal tumescence
Jurke et al. (2001)	E1G and E1S and Progestin metabolites ( + Cr)	RIA, HPLC, GC‐MS Urine and fecal samples collected “near daily” Captive	Urinary estrone metabolites are a good indicator of cyclicity (fecal are not) Extended maximal tumescence masks timing of ovulation Adult males tend to copulate around time of peak estrogen
Reichert et al. (2002)	iPd	EIA Fecal samples collected 4–7/week Captive	In 30% of cycles the presumed day of ovulation did not fall within the period of maximum tumescence Swellings in bonobos provide honest information on the probability of ovulation, but not its exact timing
			Sexual behavior of both sexes increased with degree of the swelling but not around the time of ovulation
Douglas et al. (2016)	E1 and Pd ( + Cr)	LC‐MS/MS Urine samples every 2–3 days Field	Cycle length average of ~ 40 days Bonobos can resume swellings 3 months after parturition Anovulatory cycles discovered (swelling, no ovulation)
			Supported conclusion that in 30% of cycles ovulation occurred outside of maximal tumescence
Hashimoto et al. (2022)	E1C and PdG ( + Cr)	EIA “Irregular” urine samples Field	Determined post‐partum timing of resumption of sexual swellings, copulation, ovulation, infertility, and weaning period
This article	E2 ( + Cr)	EIA ‘Irregular’ urine samples Captive	Urinary E2 can also be used to track ovulation Irregular sampling may make differentiating reproductive status more challenging
			Maximal tumescence still useful in estimating relative ovulatory status

Abbreviations: Cr, creatinine; E1, oestrone; E1C, estrone conjugates (primarily estrone‐3‐glucuronide); E1G, estrone‐glucuronide; E1S, estrone sulfate; EIA, enzyme immunoassay; GC‐MS, gas chromatography‐mass spectrometry; HPLC, high pressure liquid chromatography; iPd, immunoreactive pregnanediol; LC‐MS/MS, liquid chromatography‐tandem mass spectrometry; Pd, pregnanediol; PdG, pregnanediol glucuronide; RIA, radio immunoassay.

Although researchers have been studying urinary estrogens in primates for many years now, only a few heavily studied species have attained a true depth of endocrinological data and knowledge. While basic biological functions of estrogen remain conserved between species, its patterns of excretion and behavioral functions do appear to vary between species, such as the potentially different functional role of hormones like androstenedione or estradiol in female dominant species such as ring‐tailed lemurs (*Lemur catta*) (Drea 2007). Bonobos, known for their own female dominance system, have had relatively minimal endocrinological data published on them despite the possibility of unique or differential estrogen patterns/functions. While the endocrine pattern of ovarian cycles in captive bonobos has been described using various estrogen correlates, research on this topic is still scarce and none of these studies have examined whether directly measuring estradiol present in urine samples is both a feasible and useful means of elucidating these patterns in bonobos. Our current study contributes to this body of literature by examining the utility of measuring variation in urinary estradiol, specifically that available from highly irregularly collected urine samples, and determining the potential usefulness of this methodological approach to future reproductive monitoring or behavioral endocrinology work.

Although the best protocols for urine collection and hormone analyses involve equal and regular sampling and controlling for time of day, this is often not possible in zoo and field settings where samples must be collected opportunistically. Opportunistic sampling presents the least amount of disruption to the lives of the captive animals and the valuable time of the animal care staff. Noninvasive sampling and hormone monitoring has been shown to provide a stress‐free means of accurately evaluating reproductive status in primates (Shimizu 2005). Being able to monitor reproductive hormones with reliability, accuracy, and minimal effort can contribute significantly to behavioral, reproductive, and health management goals. Such reproductive monitoring has been used to detect changes in reproductive status, changes in fertility, and signal hormonal imbalances that are known to be risk factors for a number of health conditions in many primates including bonobos (Coburn et al. 2019; Jurke et al. 2000; Shimizu 2005). Understanding the complex variation of a naturally cycling hormone like estradiol is complicated by the limited sample availability afforded by irregular, opportunistic sampling as well as by the relatively small sample size of adult female bonobos available at any given time in each reproductive state.

The goal of this study was to better understand the complex nature and potential limitations of studying a naturally cycling hormone like estradiol in contexts in which only opportunistic sampling is available. We assessed estradiol levels of adult female bonobo urine samples collected opportunistically by zoo staff over multiple years at the Columbus Zoo and Aquarium (CZA) to test whether estradiol variation present in a data set of this nature is reliably related to reproductive status, swelling size, age, and/or social rank. Our hypotheses and predictions for this study are based on known estradiol variations in human and nonhuman primates (Graham 2012; Hashimoto et al. 2022; Saltzman et al. 2011), thus confirmation or rejection of these predictions should reflect the validity of the data set created. We tested four hypotheses:

1. *Reproductive status*: Estradiol should exhibit measurable differences between reproductive states. Urinary E2 levels should be consistently highest for pregnant females, lowest in lactating females, and exhibit intermediate, fluctuating levels in nonpregnant, nonlactating adult females.
2. *Sexual swelling:* Estradiol should vary with the ovulatory cycle and the size of sexual swelling. We predict that urinary E2 measurements of regularly cycling females (i.e. nonpregnant, nonlactating) will be highest at maximal tumescence.
3. *Age:* Estradiol varies with age over the scale of a lifespan, with levels declining as females approach old age and potential menopause. We predict that, given sufficient age variation in the study population, peri‐menopausal and older females will show lower urinary E2 levels.
4. *Rank and individual differences:* As other studies have not reported significant differences between subjects in terms of overall levels of urinary estrogen metabolites, we predict minimal between subject differences in estradiol levels, with most differences being explained by reproductive status, swelling size, and age, and not individual factors such as social rank.

## 2 Methods

### 2.1 Subjects and Housing

Subjects for this study included four adult female bonobos housed at the CZA in Columbus, Ohio, USA. Samples were collected from these individuals during the summer months of 2012, 2013, 2014, and 2015, with the first samples being collected in May 2012 and last in August 2015. Over the course of the study, group composition at the CZA included a total of 11 females (4 adult, 7 subadult) and 8 males (5 adult and 3 subadult) who were group‐housed in a complex of indoor and outdoor enclosures. The social groups were in continuous full social contact within these enclosures; additionally, CZA keepers attempted to simulate the species typical fission‐fusion process of variable party composition by allowing the bonobos access to each other every morning and establishing subsequent parties based mostly on individual bonobo association preferences (see Boose et al. 2013 for more description on group housing scheme). At the beginning of data collection for this study in 2012, Unga was the youngest bonobo at 19 years old, Ana Neema was 20, and Susie and Lady were both 30 years old.

### 2.2 Determining Reproductive and Rank Status of Females

All of the adult females in this group were multiparous and three of these females either gave birth, were pregnant, or were currently breastfeeding an infant ( ≤ 2 years of age) over the course of this study. Pregnancy status was known and confirmed by CZA staff and thus applied to all samples during which a female was known to be pregnant. Following the birth of an infant, females were presumed to be in lactational amenorrhea and classified as such when they were seen nursing an infant while showing very little variation in size of sexual swellings. Sexual swelling ratings were reported for all the females on a daily basis by two different CZA keepers. Sexual swellings were scored on a scale of 0–3 based on degree of maximum tumescence as defined for bonobos by previous research (Dahl 1986; Douglas et al. 2016).

The relative dominance rank order of the four females in the group remained consistent over these 4 years of study with Unga consistently ranking as the dominant female, followed by Ana Neema, Susie, and then Lady. In 2012 the third dominance rank position among females was supplanted by an adolescent female, JoT (who was not included in this study) but was reclaimed by Susie after JoT left the group in 2013; the relative positions among the four adult females in this study remained consistent throughout this process. Rank determinations for these individuals have been established by previous studies (Boose et al. 2018; Boose and White 2017) and were based on the direction and outcome of decided agonistic events.

### 2.3 Urine Collection and Storage

Urine samples were collected by CZA staff either by using a free catch method in which individual subjects urinated through the mesh caging directly into a collection cup, or, by pipetting urine directly off a clean floor surface into a plastic cryo‐tube. All subjects had received previous training to urinate on command in exchange for a small food reward. Variability in sample quantities and availability was present between subjects due to the nature of opportunistic sample collection and the fact that some of the females were known to provide more consistent urine samples than others. Opportunistic sampling involves the collection of urine samples when available, as is often the case in zoo and wild research contexts, as opposed to the regular hourly or daily sampling that may be used to establish hormone profiles in a laboratory setting. Opportunistic sampling at the CZA has been sufficient to establish hormone profiles for other hormones including cortisol, oxytocin, and testosterone in this study population (Boose et al. 2018; Boose and White 2017).

Upon collection, samples were immediately frozen and stored at − 20°C until they were packed on dry ice and shipped overnight to the Global Health Biomarker Lab at the University of Oregon in Eugene, OR where they were stored at − 80°C until time of analysis. While the number of freeze‐thaw cycles the samples have undergone has been minimized whenever possible, many samples used in this study have undergone multiple freeze‐thaw cycles since being collected due to prior analyses for previous publications (Boose et al. 2018; Boose and White 2017). The efficacy of frozen urinary analytes over extended periods of time has been validated by numerous studies and steroid hormones have been found to be very stable in both plasma and urine even when frozen for durations longer than 10 years (Fuhrman et al. 2010; Kesner et al. 1995; Kley et al. 1985; Miki and Sudo 1998; Moffat et al. 2020). Urinary creatinine is known to be particularly stable and virtually unaffected by storage time and temperature (Spierto et al. 1997).

### 2.4 Measurement of Estradiol

For analysis, samples were brought to room temperature and diluted (1:4) in assay buffer supplied in the 96‐well Detect X Estradiol ELISA Kit from Arbor Assays (catalog no. K030‐H) and assayed according to the kit manufacturer's instructions. The manufacturer of this kit reported the detection limit for this assay as 39.6 pg/mL and cross‐reactivity of 100% with 17β‐estradiol, 0.78% with estrone, 0.22% with 17α‐estradiol, 0.11% with 17α‐ethynylestradiol, and less than 0.10% with all other potential correlates assessed. Each plate allows 40 samples to be run in duplicate. This Arbor Assays ELISA protocol was specifically designed to be used with urinary and fecal samples, and it has been validated for use in a wide array of human and animal models.

Additional validation tests for parallelism and spike recovery were conducted to ensure the accuracy of this assay in bonobo urine in particular as this was the first time this kit was used for this species. For the test of parallelism, two‐fold serial dilutions of four samples with high but readable endogenous estradiol concentrations were run in duplicate along with neat samples on the same plate. The concentrations obtained were appropriately compensated for differences in dilution factor and the %CV of the concentration values generated for each sample were calculated. For the spike recovery test, three pooled samples were diluted (1:4) then spiked using known quantities of estradiol standard solution to three levels across the standard curve. The recovery percentage of each spiked sample was calculated by comparing the measured concentration of the spiked sample to theoretical, expected concentration values.

Controlling for urinary creatinine levels is a validated means of correcting urinary hormone levels for concentration differences between samples (Hodges et al. 2017; Kinoshita et al. 2016; Miller et al. 2004). All of our samples were assayed for creatinine concentrations using DetectX Urinary Creatinine Detection Kits from Arbor Assays (catalog no. K002‐H). The manufacturer listed the detection limit for this assay as 0.019 mg/dL. Samples run for creatinine were diluted (1:20) and run according to the kit manufacturer's instructions. This methodology and assay kit have previously been validated for use with bonobo urine (Boose et al. 2018). All plates were read using a BioTek microplate reader and analyzed with Gen5 software version 2.0.

### 2.5 Data Analyses

Of the frozen urinary samples available, we were able to successfully assay 31 samples from Ana Neema, 16 from Lady, 48 from Susie, and 22 from Unga for a total of 117 samples included in our analyses. The estradiol concentration value of each sample was divided by its respective creatinine value to generate estradiol levels corrected for differences in urinary concentration; all subsequent references to estradiol levels in the results, figures, and conclusion can be assumed to be referencing estradiol levels that have been corrected using creatinine concentration. As the corrected estradiol concentrations obtained were not normally distributed, the data was log 10 transformed and all groups were determined to be not significantly different to normal as assessed with a Kolmogorov‐Smirnov test.

ANOVAs were used to compare log‐transformed estradiol levels between different reproductive statuses (pregnant, lactating, and regularly cycling). We ran a two‐way ANOVA with individuals fit as a random factor and reproductive status fit as a fixed categorical effect to determine if individuals could be combined within reproductive statuses in this unbalanced sampling distribution. Data were subsequently categorized by reproductive status and analyzed separately. Regressions were used to assess the relationship between estradiol levels and same‐day sexual swelling ratings within each reproductive status. The amount of variation in estradiol due to sexual swelling was then statistically removed and the residuals between females were compared with Pearson correlations to examine the relationship between estradiol concentration, rank, and age within these regularly cycling females. We used an alpha level of 0.05 for all statistical tests. All statistics were run in SAS^(c)^ statistical software version 9.2 (SAS Institute 1992).

## 3 Results

Methodologically, our assay results confirmed that estradiol can be reliably detected in bonobo urine using this ELISA protocol and kit. Of a total of 130 samples that we attempted to assay, only 13 samples were not included in the final analysis because they had a coefficient of variability (%CV) between duplicate wells beyond our threshold of 20%. These samples could not be re‐run given insufficient quantities of remaining urine samples. Of the 117 samples included in our final analysis, we calculated an average intra‐assay %CV of 8.2% and an average inter‐assay %CV of 3.98% between the four plates run. These findings indicate high consistency of measurement both within and between the plates. Sufficient levels of parallelism (%CV less than 30%) were reported in the four serially diluted samples and the three spike recovery tests resulted in a mean recovery of 87.50%; both validation test results fall within acceptable ranges reported by previous studies and serve to further validate the accuracy of results collected from bonobo urine using this assay kit (Andreasson et al. 2015).

**Figure 1:**
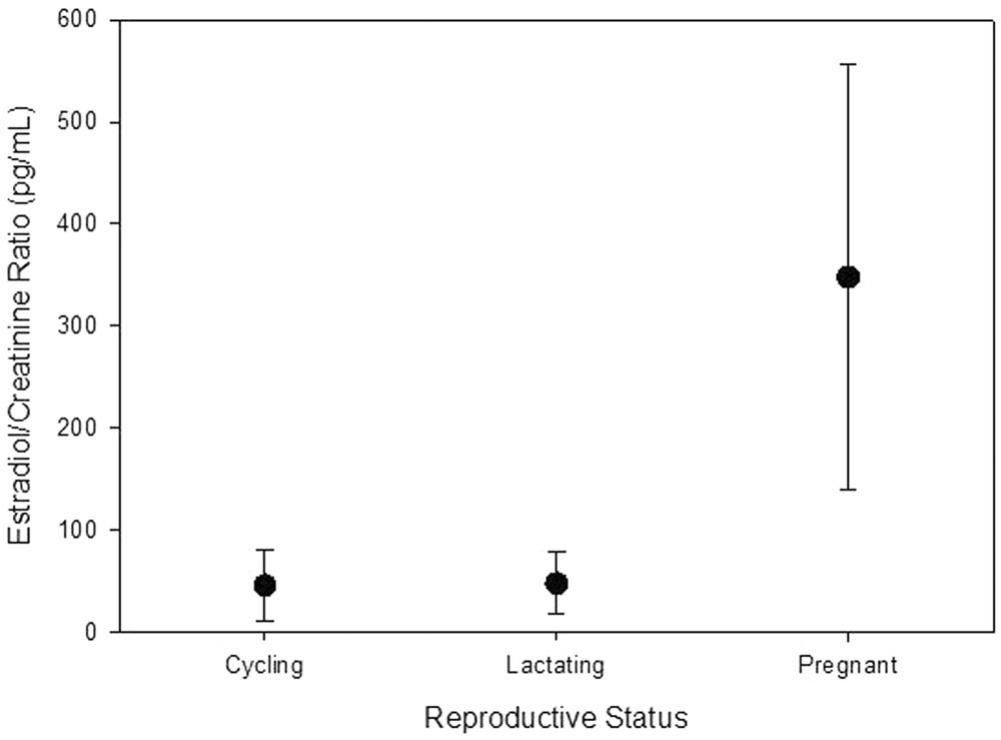
Estradiol/creatinine ratio means with standard deviations, compared between reproductive states (regularly cycling, lactating, and pregnant). There was overall significance detected with post hoc analysis revealing that the significance was driven by the difference between pregnant and nonpregnant females.

Our two‐way ANOVA was significant (F = 11.10, *df* = 8, 108, *p* < 0.0001) and specifically revealed a significant effect of reproductive status on estradiol levels (F = 41.43, *df* = 2, 108, *p* < 0.0001) but no effect of individuals (F = 0.63, *df* = 3, 108, *p* = 0.5991) and no interaction term between reproductive status and individuals (F = 1.36, *df* = 3, 108, *p* = 0.2605). As individuals were not significantly different, we subsequently pooled within each reproductive status and confirmed that estradiol levels were significantly different between the three reproductive statuses (pregnant, lactating, or neither) (F = 41.45, *df* = 2, 114, *p* < 0.001). A closer examination of this result using a priori multiple comparisons (Sokal and Rohlf 2012) revealed that estradiol in pregnant females was significantly higher than in all nonpregnant females (F = 68.11, *df* = 1, *p* < 0.001) and not significantly different between lactating and regularly cycling females (F = 0.27, *df* = 1, *p* = 0.6041) (see Figure 1).

Sexual swellings exhibited minimal variation in pregnant and lactating individuals, being mostly of the smallest size categories, so we compared estradiol levels across only samples of “small” swelling sizes (0–1) and found that there was still a significant effect of reproductive status on estradiol levels even when swelling sizes were equivalent (F = 39.64, *df* = 2, 71, *p* < 0.001). A closer examination of this result using a priori multiple comparisons found there was significance between pregnant and nonpregnant females (F = 66.30, *df* = 1, *p* < 0.001) but not between lactating females and regularly cycling females at the lowest sexual swelling level (F = 0.40, *df* = 1, *p* = 0.5304).

**Figure 2:**
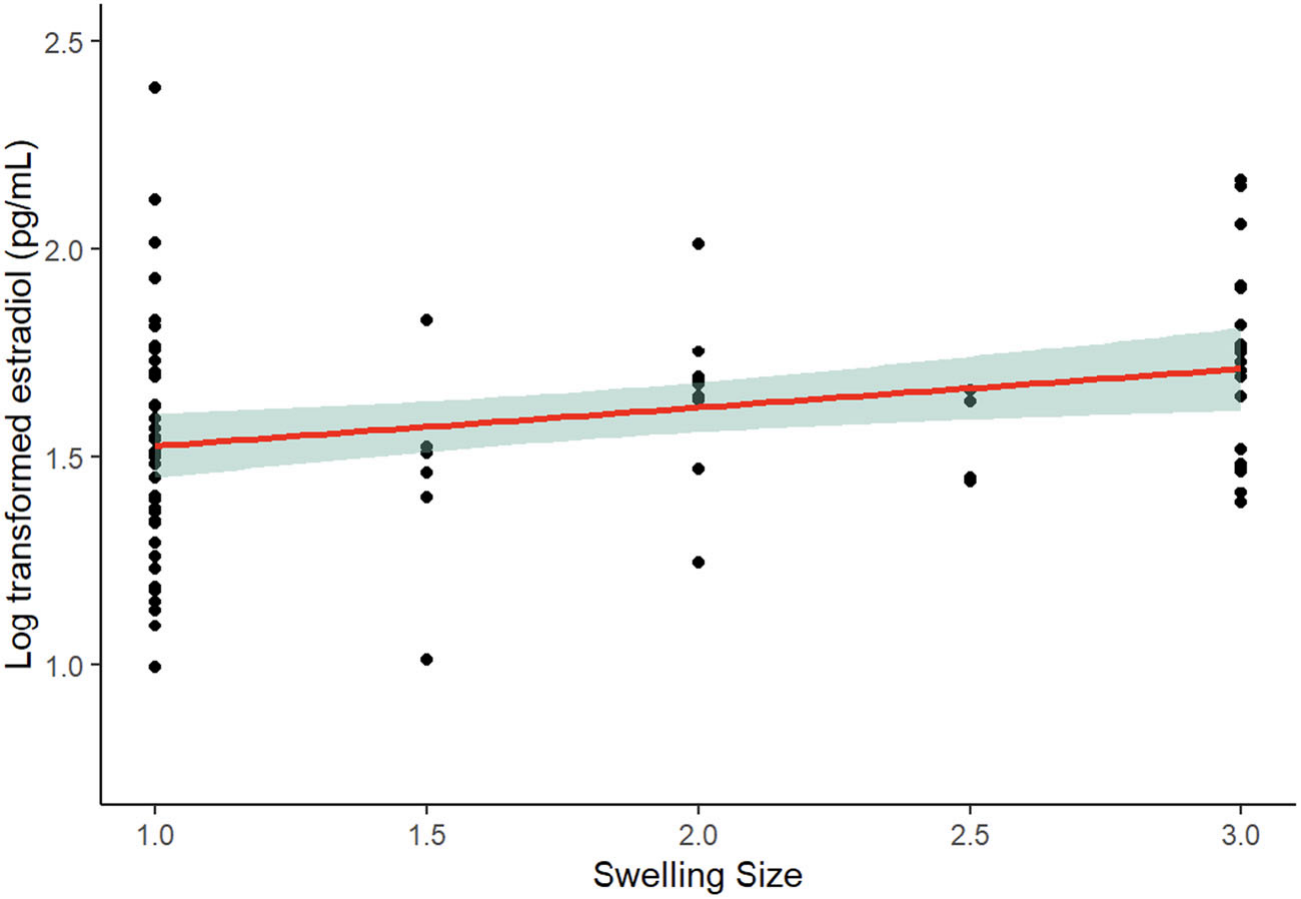
Log‐transformed estradiol values compared between different swelling size categories (1–3) in regularly cycling females. Estradiol increased with higher swelling ratings.

For the regularly cycling females, who did exhibit greater variation in swelling size, there was a significant positive regression between estradiol and swelling size (F = 4.43, *df* = 1, 81, *p* = 0.0384) (see Figure 2). We used the residuals from this regression to statistically remove the relationship of sexual swelling on estradiol so that the further comparisons below could examine individual differences without being complicated by differences in sexual swelling size.

When the amount of variation in estradiol due to sexual swelling was statistically removed for regularly cycling females, as above, and the residuals between females were compared, there was no significant effect of age (*n* = 83, r = 0.08059, *p* = 0.4689) or rank (*n* = 83, r = 0.1361, *p* = 0.22) on estradiol levels for these females.

## 4 Discussion

The results of this study showed that measurement of excreted estradiol levels in urine was a valid means of detecting expected estrogen trends in bonobos. While urinary estradiol levels in opportunistically collected samples were significantly different between pregnant and nonpregnant female bonobos, they were not capable of differentiating between lactating and regularly cycling females. This irregular data set showed that as sample sizes get smaller, only the broadest of hormonal changes may be detected, such as those between pregnant and nonpregnant females.

Our initial analyses revealed that reproductive status was the primary driver of differences in estradiol levels seen in this study group. This highlights the importance of knowing the reproductive state when interpreting estradiol data. The overlap in estradiol levels between the three stages was sufficiently large to indicate that a single estradiol level should not be used to definitively determine the reproductive status of individual, but a larger sample of estradiol levels over time could be used to predict or confirm the reproductive status of a bonobo in the field with confidence limits. While the result that estradiol levels were higher in pregnant females reflects known estradiol patterns, the fact that samples taken from the lactating females in this study were not lower than those of regularly cycling females was surprising. While bonobos tend to be in lactational amenorrhea for approximately 24 months following birth, they are also known to have a disconnect between the timing of the resumption of sexual behaviors, actual hormonal estrus cycles, and visual changes in sexual swellings (Hashimoto et al. 2022). Given the fact that bonobos are sometimes capable of giving birth after a relatively short interval, such as having second infant while still nursing a first (Furuichi and Hashimoto 2002) it is possible that some of the females we presumed to have been in lactational amenorrhea may have resumed ovulation earlier than expected and while they were still nursing their previous infant. Additionally, it is possible that an irregularly sampled data set may fail to capture days of ovulation, artificially lowering the average estradiol values for regularly cycling females. The high degree of overlap we discovered among individuals' cycling and lactating estradiol values indicates that one potential shortcoming of a highly opportunistically sampled data set of this kind is that these two reproductive states cannot be reliably differentiated. In the previously published bonobo estrogen profiles, while there are often a few days with particularly high estrogen values around the time of ovulation, there is usually 1 day in particular with a notable estrogen peak (Heistermann et al. 1996; Jurke et al. 2001; Reichert et al. 2002). Any non‐daily sampling scheme interested explicitly in tracking ovulation should account for the potential of missing the day of peak estrogen levels.

As bonobo sexual swellings are always relatively large, and exhibit extended maximal tumescence, it was anticipated that visual assessment and rating of bonobo sexual swellings may be uniquely challenging such that swelling size may not map well on to higher/lower estradiol levels. Previous studies of sexual swellings in bonobos have also indicated that visual tumescence is not the most reliable indicator of fertility in bonobos (Douglas et al. 2016; Hashimoto et al. 2022; Thompson‐Handler 1990). However, this study found that larger swelling ratings were consistently associated with higher levels of estradiol, thus showing that sexual swellings were communicating useful information regarding relative estradiol levels and ovulatory status in these females. The clarity of this result may have been the result of the use of a relatively abbreviated set of size classes (0–3), rather than a more detailed division into many size classes (e.g. 0–7) which may track less explicitly onto differences in estradiol levels; it is not clear how much sexual swelling varies on a day‐to‐day or even hour‐to‐hour basis. The simple system used here, however, was associated with estradiol levels and serves to validate the rating system employed by CZA keepers and more generally the use of visual assessment of tumescence as a means of estimating cycle stage in bonobos.

Age was not found to significantly affect estradiol levels in these females. The age range of the females in this study group was twelve years and did not include either very young or very old females and thus may not have been large enough to capture the age‐related increases and reductions in estradiol levels that occur over a full lifetime. In addition, this study only captured 4 years of estradiol levels which might also be insufficient to show a lifetime trend as bonobos live for 40–50 years (Rowe 1996). We might have expected to see an effect of age had there been any post‐menopausal females in the population. The presence of menopause in female bonobos remains unconfirmed. Limited hormonal evidence of the disruption of hormonal cycles has been observed in captive 40 + year old bonobos (Gould et al. 1981) as well as behavioral evidence of the cessation of visual cycling and reproduction in wild populations (White and Churchill 1997), but conclusive hormonal evidence of human‐like menopause has thus far only been reported in chimpanzees (Wood et al. 2023).

The four females in this study did not differ significantly in their estradiol patterns and exhibited the same hormonal responses to changes in reproductive status and with swellings across the ovulatory cycle. There was no effect of social rank on variation in estradiol levels detected in this study. This lack of a correlation indicates that if there is any relationship between social status and estradiol levels it is more nuanced than could be detected with this data set.

Regularly monitoring hormone levels allows animal managers to establish expectations, detect when changes occur, and interpret those changes correctly. The more samples you have, the better your ability to detect deviations will be. Given the degree of variability in postpartum infertility periods of bonobos and the decoupling of the resumption of sexual cycles, sexual swellings, and sexual behaviors having regular estrogen monitoring in place can provide vital management information. Biological evidence can now be paired with visual information to help guide decisions regarding housing and mating opportunities. Developing techniques for more regular urine collection, whether that be through training zoo populations or developing field techniques such as the drying of urine on filter paper (Knott 2005; Mouri and Shimizu 2021; Shideler et al. 1995), will become necessary for researchers interested in establishing useful collections of hormonal data in these settings.

For researchers interested in the behavioral endocrinology of bonobo behaviors, once baselines for estradiol levels in bonobos at different reproductive phases are established, hormonal data sets can be paired with temporally synced behavioral data to study more situation‐dependent estradiol fluctuations and behavioral interactions. Estrogens tend to be under‐studied in behavioral contexts as compared to hormones like cortisol, oxytocin, or testosterone, which may be easier to monitor and have more obvious behavioral functions. Such research contributes to the development of models for the potential impacts and feedback loops present between estradiol and behavior in much the same way the challenge hypothesis has helped us understand the relationship between testosterone and aggression (Wingfield et al. 1990; Muller and Wrangham 2004). Currently much more research in the field of socio‐endocrinology is being devoted to questions surrounding the social functionality of testosterone in male primates, while comparatively less is known about the behavioral effects of estrogens. Undoubtedly there are biases at play in terms of the questions being asked in the field of human and nonhuman primate behavioral endocrinology, with one review article of human endocrinology research reporting that there is nearly two to three times the amount of research being done on hormones and competition in men as opposed to in women (Casto and Prasad 2017).

Despite the complexities inherent in studying sources of variation in estradiol and its various biological and behavioral capacities within the body, establishing accessible methodologies for its study, particularly in less studied species, will continue to be a vital contribution to this field. The more information we have on different ways to measure the hormone and factors that may influence its levels the better we will be able to interpret and contextualize what data is available. In publishing more results of this kind and providing details on the methodological approach taken, we hope to provide data that will benefit other researchers with similar intellectual interests and methodological constraints.

## Author Contributions

**Sara Cotton:** conceptualization (equal), formal analysis (equal), funding acquisition (lead), methodology (equal), visualization (equal), writing – original draft (lead), writing – review and editing (equal). **Klaree Boose:** conceptualization (supporting), methodology (equal), writing – review and editing (supporting). **Sedona Espstein:** visualization (equal), writing – review and editing (supporting). **Audra Meinelt:** resources (supporting), writing – review and editing (supporting). **Josh Snodgrass:** resources (supporting), writing – review and editing (supporting). **Frances White:** conceptualization (equal), formal analysis (equal), writing—review and editing (equal).

## Ethics Statement

All protocols and procedures implemented in this study were ethically reviewed and approved by the University of Oregon Institutional Animal Care and Use Committee, adhered to the American Society of Primatologists (ASP) principles for the ethical treatment of nonhuman primates, and were in compliance with all other legal regulations for research in the United States.

## Data Availability

The data that support the findings of this study are openly available in Dryad at https://doi.org/10.5061/dryad.v6wwpzh4q. All deidentified data and metadata resulting from this project will be archived in Dryad (Cotton 2025). Pre‐prints of papers resulting from this study will be archived in the UO Scholar's Bank institutional repository with DOI links to their associated data in Dryad. Original paper files will be maintained in locked file cabinets in locked storage facilities for up to 10 years, then placed in long‐term archival facilities or shredded. In the event that the PI leaves UO, the UO will work with the PI, per UO policy, to transfer active grants while retaining data as necessary to meet the state of Oregon's record retention policy. If the data is not part of an active grant being transferred, UO will work with the PI's new institution to develop an appropriate data use agreement.
